# Closing Editorial: Subjective Time—Cognition, Emotion and Beyond

**DOI:** 10.3390/ejihpe16070104

**Published:** 2026-07-21

**Authors:** Quentin Hallez, Sandrine Gil

**Affiliations:** 1 Unité de Recherche DIPHE “Développement, Individu, Processus, Handicap et Éducation”, Institut de Psychologie, Université Lumière Lyon 2, 69500 Bron, France; 2 Centre de Recherches sur la Cognition et l’Apprentissage (CeRCA), Université de Poitiers, Université de Tours, CNRS, 86000 Poitiers, France; sandrine.gil@univ-poitiers.fr

## 1. Introduction

Time is a peculiar object for psychological science. Although it structures every action, decision, and interaction, it is never given to us directly: it must be reconstructed by the cognitive system, colored by emotion, and interpreted through the lens of individual histories and social contexts. Research on subjective time has traditionally developed along two largely independent lines. The first, rooted in the psychophysics of timing and in internal clock models (Gibbon et al., 1984), investigates how durations of seconds and minutes are perceived, estimated, and reproduced, and how cognitive resources constrain these operations. The second, stemming from the time perspective tradition (Zimbardo & Boyd, 1999), examines the habitual ways in which individuals relate to their past, present, and future, and how these orientations shape motivation, health, and social behavior.

The Special Issue “Subjective Time: Cognition, Emotion and Beyond” was launched with the ambition of bringing these two levels of analysis into dialogue. We invited contributions examining how we experience and perceive time, with particular attention to the interplay between cognitive mechanisms and emotional processes, across different populations and stages of the lifespan. Five original articles were ultimately published in this collection. Remarkably, although they span very different populations—children with and without neurodevelopmental vulnerabilities, university students, young and middle-aged adults from several cultural contexts—and very different methods, they compose a coherent picture that can be organized around two complementary axes: measured time, or how the cognitive cost of processing durations can be either endured by the individual or absorbed by a well-designed environment (Section 2.1); and time perspectives as drivers of personal and social adaptation (Section 2.2).

## 2. An Overview of the Published Contributions

### 2.1. Measured Time: From an Endured Cognitive Load to a Supportive, Designed Environment

Two contributions converge on a central idea: processing temporal information is cognitively expensive, and this cost is not distributed equally across individuals—but it can be substantially alleviated by the environment.

On one side, Gourlat et al. (contribution 1) document just how costly temporal processing can be. Comparing 79 young people aged 10 to 20 years with idiopathic mild intellectual disability to 81 typically developing peers on a temporal reproduction task, together with a battery of executive and speed measures, they show that duration estimation deficits are largely explained by domain-general cognitive functions. Specifically, slower information processing speed accounted for most of the deficit in the accuracy of temporal reproductions, whereas weaker inhibition abilities accounted for most of the increased variability of responses. In other words, when one must rely exclusively on one’s internal clock, the precision and stability of temporal judgments are directly bounded by the efficiency of the cognitive resources available to feed and monitor that clock.

On the other side, Hallez and Vallier (contribution 2) show what happens when this cognitive load is externalized by making time visible. In their study, 44 children aged 7 to 9 years completed a timed mathematics assessment with and without a visible Time-Timer. The presence of the visual timer produced a significant reduction in anticipatory evaluation anxiety measured before task onset, as well as a significant decrease in inattentive and motor-instability behaviors during the task—an effect that was most pronounced in children at higher risk for Attention Deficit Hyperactivity Disorder (ADHD). Interestingly, engagement with the device was highly heterogeneous, with a quarter of the children consulting it more than seven times in five minutes, reminding us that environmental supports also interact with individual differences in how they are used.

Read together, these two contributions suggest that time is a stressful variable when one depends solely on one’s internal clock, especially in the presence of executive vulnerabilities, but that an adequate environmental design may allow individuals to reallocate their cognitive resources toward the task itself rather than toward timekeeping. This convergence invites a broader reflection. Crossing the findings on intellectual disability and on children at risk for ADHD, subjective time clearly emerges as a potential barrier to inclusion: the very populations for whom temporal processing is most costly are also those most exposed to timed, standardized situations. One may therefore ask whether temporal ergonomics, such as the use of visual timers, should not be conceived as a universal accessibility tool, built by default into our standardized assessments and school environments rather than offered as an individual accommodation.

### 2.2. Time Perspectives as Drivers of Personal and Social Adaptation

The three remaining contributions move from the perception of durations to the broader psychological relationship individuals entertain with time, and show that this relationship operates as a genuine adaptive resource (or barrier) in three domains: academic engagement, mental health, and altruism.

Regarding academic engagement, Mathieu et al. (contribution 3) used latent profile analysis on 451 French first- and second-year university students and identified five time perspective profiles based on the Zimbardo Time Perspective Inventory. A balanced time perspective profile, gathering a third of the students, was the profile most strongly associated with study engagement, whereas profiles marked by a negative view of the past displayed the lowest engagement scores. Considering profiles rather than isolated temporal orientations, the authors argue, offers a promising way to detect students who most need support during their studies.

Regarding mental health, Bashkin et al. (contribution 4) examined attitudes toward seeking mental health services in Russian Y (*n* = 217) and Z (*n* = 256) generations. Our relationship to the present, their results show, partly dictates our coping strategies: a fatalistic present orientation, a feeling of helplessness and limited control over one’s life, was a negative predictor of help-seeking attitudes in both generations. Contrarily, personal growth was a positive predictor, complemented by generation-specific predictors (positive relations and hedonistic present in Generation Y, future orientation in Generation Z).

Regarding altruism, Niiya et al. (contribution 5) demonstrate across three studies (i.e., one correlational and two experimental) that the perception of time directly influences our behavior toward others. Perceiving time as a “nonzero-sum” resource, that is, as a resource created moment-by-moment in our interactions rather than lost when given away, significantly increased participants’ willingness to help, an effect mediated by the prosocial motivation to reduce others’ distress. Time spent helping another person, under this construal, is no longer time lost for oneself but a resource invested for the benefit of both.

Here as well, a reflective question emerges from the convergence of findings. If a fatalistic present hinders help-seeking, and if being stuck in a negative past undermines study engagement, should the therapeutic and educational target of tomorrow not be temporal flexibility itself? Beyond intervening on symptoms (i.e., anxiety, academic dropout, reluctance to seek help), the challenge becomes designing educational and clinical protocols that teach individuals to consciously navigate among their temporal perspectives according to the demands of the context.

Table 1 summarizes the five contributions of this Special Issue.

## 3. Conclusions

Is it possible to draw a single thread through studies ranging from the psychophysics of duration in intellectual disability to zero-sum beliefs about helping others? We believe it is, and we propose to name it temporal agency. The five contributions gathered here converge toward the same practical conclusion: human beings are not condemned to endure time. Restoring individuals’ control over their own temporality appears to be a key lever for fostering inclusion, engagement, and well-being. This control can be supported externally by arranging the physical environment so that vulnerable children devote their resources to the task rather than to timekeeping (contributions 1 and 2). It can also be cultivated internally through a balanced, flexible, and generative way of relating to one’s past, present, and future (contributions 3 to 5).

This convergence also sketches a research agenda. The dialogue between the timing literature and the time perspective literature remains embryonic, and the present collection suggests it would be fruitful. Indeed, the cognitive resources that constrain duration processing and the motivational orientations that shape our relationship to time are likely to interact, particularly in populations with executive vulnerabilities. Longitudinal and interventional designs are now needed to test whether temporal ergonomics and training in temporal flexibility produce lasting benefits, and for whom. We hope that this Special Issue will encourage researchers and practitioners alike to treat subjective time not merely as a variable to be measured, but as an environment to be designed and a competence to be nurtured.

We warmly thank the authors of the five contributions for the quality of their work, the reviewers for their constructive expertise, and the editorial team of the *European Journal of Investigation in Health, Psychology and Education* for its support throughout the preparation of this Special Issue.

## Figures and Tables

**Table 1:** Overview of the contributions published in the Special Issue “Subjective Time: Cognition, Emotion and Beyond”.

**Contribution**	**Population**	**Temporal Construct**	**Main Finding**
1. Gourlat et al.	Youth (aged 10–20) with mild intellectual disability vs. typical development	Duration estimation (temporal reproduction)	Processing speed accounts for accuracy deficits; inhibition accounts for response variability
2. Hallez & Vallier	Children aged 7–9 in timed math assessment	Externalized time (visual timer)	Lower anticipatory anxiety; fewer inattentive and motor-instability behaviors, especially at higher ADHD risk
3. Mathieu et al.	French university students (*N* = 451)	Time perspective profiles	Balanced time perspective profile shows the highest study engagement; past-negative profiles the lowest
4. Bashkin et al.	Russian Y and Z generations (*N* = 473)	Time perspective and well-being	Fatalistic present hinders, and personal growth promotes, attitudes toward seeking mental health services
5. Niiya et al.	Adults (three studies, correlational and experimental)	Zero-sum vs. nonzero-sum time perception	Perceiving time as nonzero-sum increases willingness to help, mediated by prosocial motivation
